# Synthesis and Use of Valsartan Metal Complexes as Media for Carbon Dioxide Storage

**DOI:** 10.3390/ma13051183

**Published:** 2020-03-06

**Authors:** Alaa Mohammed, Emad Yousif, Gamal A. El-Hiti

**Affiliations:** 1 Department of Chemistry, College of Science, Al-Nahrain University, Baghdad 64021, Iraq; alaaalqaycy7@gmail.com; 2 Cornea Research Chair, Department of Optometry, College of Applied Medical Sciences, King Saud University, P.O. Box 10219, Riyadh 11433, Saudi Arabia

**Keywords:** valsartan metal complexes, carbon dioxide storage media, adsorption capacity, pore size, surface area, porous materials

## Abstract

To address global warming through carbon dioxide storage, three valsartan metal complexes were synthesized in excellent yields (87–92%) through a reaction of the appropriate metal chloride (tin chloride, nickel chloride hexahydrate, or magnesium chloride hexahydrate) and excess valsartan (two mole equivalents) in boiling methanol for 3 h. The structures of the metal complexes were established based on the data obtained from ultraviolet-visible, Fourier transform infrared, and proton nuclear magnetic resonance spectra, as well as from elemental analysis, energy-dispersive X-ray spectra, and magnetic susceptibility. The agglomeration and shape of the particles were determined using field emission scanning electron microscopy analysis. The surface area (16.63–22.75 m^2^/g) of the metal complexes was measured using the Brunauer-Emmett-Teller method, whereas the Barrett-Joyner-Halenda method was used to determine the particle pore size (0.011–0.108 cm^3^/g), total average pore volume (6.50–12.46 nm), and pore diameter (6.50–12.47 nm), for the metal complexes. The carbon dioxide uptake of the synthesized complexes, at 323 K and 4 MPa (40 bar), ranged from 24.11 to 34.51 cm^2^/g, and the nickel complex was found to be the most effective sorbent for carbon dioxide storage.

## 1. Introduction

Earth’s atmosphere acts as a solar energy snare to maintain the global temperature at a natural average level to make life on earth possible. The atmosphere tends to absorb heat and re-emit the energy back into space in a different direction [1]. Over the years, the atmosphere, seas, and oceans have stored billion of tons of carbon dioxide (CO_2_) in the process of establishing the carbon cycle [2], but the high demand for energy has led to the consumption of fossil fuels on a very large scale. As a result, high levels of greenhouse gases such as CO_2_ are produced in the environment, leading to global warming [3,4,5,6]. The current high level of CO_2_ upsets the natural balance of the solar system on Earth. Such effect leads to an increase in the Earth’s temperature, floods due to rising sea levels, the melting of ice at the South and North poles, and undesirable weather changes [7]. In addition, natural gases containing CO_2_ have poor quality and energy capacity and could corrode pipelines [8]. Therefore, lowering the CO_2_ level in the atmosphere has become a priority. Two convenient routes can be followed simultaneously: either use renewable sources of energy, which are currently not sufficient, or develop new technologies to capture and store CO_2_ [9,10,11]. Recently, the design and use of new materials to capture and store greenhouse gases has become a hot topic [12,13,14,15].

CO_2_ uptake and storage is a technology that is commonly used to decrease the level of CO_2_ in the atmosphere [14]. A process using ethanolamine as an adsorbent for CO_2_ has proven to be expensive to implement, because it requires high energy and the use of highly volatile materials [16,17]. Therefore, alterative chemical adsorbents are needed that are simple and cheap to produce, making the process of adsorption economically viable [18]. Materials used to adsorb CO_2_ should have an excellent adsorption capacity and the ability to be regenerated and reused several times without losing their efficiency [19]. Zeolites, silica, and activated carbon-containing materials have been tested as storage media for CO_2_ [20,21,22,23,24]. However, little success has been achieved with these materials, either because they are strongly hydrophilic, as in zeolites, or they have poor gas selectivity, as in activated carbons [25,26]. Polymers, resins, and biomass have also been used to produce activated carbon materials [22]. Chemical activators can be added to activate carbon-containing materials to improve their CO_2_ uptake efficiency.

In recent years, porous materials with large surface areas have been used as CO_2_ storage media [10]. Such materials have different adsorption capacities depending on their structure [27]. The most common porous materials used in gas storage are porous organic polymers (POPs) and metal–organic frameworks (MOFs) [10,28]. Well-designed porous solids can be produced using molecular building blocks [29]. The capacity of porous MOF solids toward CO_2_ uptake can be enhanced by increasing the MOF surface area and by including polar groups within their structures [10,30]. The strong hydrogen bonds between CO_2_ and MOFs lead to high adsorption efficiencies [31]. POPs have high the needed qualities to act as effective materials to capture CO_2_ [32]. However, their synthetic procedures involve the use metal catalysts under harsh reaction conditions [25].

Valsartan, *N*-{4-[(1-(1*H*-tetrazol-5-yl) phenyl) benzyl}-*N*-valeryl-*L*-valine, is a medication that is mainly used to reduce high blood pressure and to treat heart failure [33,34]. It is highly stable, has a high molecular weight (435.5), a high aromatic content (two aryl groups and a tetrazole ring), has various functionalities (ketone, ester, and NH), and contains a high proportion (27%) of heteroatoms (nitrogen and oxygen). These properties could enable valsartan metal complexes to act as media to capture CO_2_, since POPs and MOFs are highly aromatic and contain nitrogen, oxygen, and metals (boron, silicon, and phosphorous). In the current study, the synthesis of three valsartan metal complexes and their use as media for capturing CO_2_ were investigated. Recently, we showed that various materials can be used as efficient media for CO_2_ capture [35,36,37,38].

## 2. Materials and Methods

### 2.1. General

Metal chlorides, valsartan, and solvents were purchased from Sigma-Aldrich (Schnelldorf, Bavaria, Germany). Elemental analyses of the metal complexes were performed using a Vario EL III instrument (Analysensysteme GmbH, Hanau, Germany). An AA-6880 Shimadzu atomic absorption flame spectrophotometer (Tokyo, Japan) was used to measure the metal content within the synthesized complexes. The Fourier transform infrared (FT-IR) spectra (400–4000 cm^−1^) of the metal complexes were recorded using an FT-IR 8300 Shimadzu spectrophotometer (Tokyo, Japan) and KBr pellets. The ultraviolet-visible adsorption spectra (200–800 nm) of the metal complexes were measured in ethanol using a Shimadzu UV-1601 spectrophotometer (Tokyo, Japan). Proton nuclear magnetic resonance (^1^H-NMR) spectra were measured in deuterated dimethyl sulfoxide (DMSO-d_6_) using a Bruker DRX400 NMR spectrometer (Bruker, Zürich, Switzerland) at 400 MHz. The magnetic susceptibilities of the complexes were measured using a Bruker BM6 magnetic balance (Bruker, Zürich, Switzerland). The conductivity measurements of the metal complex solutions (10^−3^ mole/L) in DMSO were performed at 25 °C on a WTW ProfiLine Oxi 3205 conventional portable meter (Xylem Inc., Weinheim, Germany). The field emission scanning electron microscopy (FESEM) and energy dispersive X-ray (EDX) analyses were performed on a TESCAN MIRA3 LMU system (Kohoutovice, Czech Republic) at an accelerating voltage of 15 kV.

### 2.2. Synthesis of Metal Complexes

A solution of the appropriate metal chloride (1.0 mmol), i.e., tin (IV) chloride (SnCl_2_), nickel (II) chloride hexahydrate (NiCl_2_·6H_2_O), or magnesium (II) chloride hexahydrate (MgCl_2_·6H_2_O), in methanol (MeOH, 5 mL) was added to a stirred solution of valsartan (0.87 g, 2.0 mmol) in MeOH (5 mL). The mixture was heated under reflux for 3 h. The solid obtained on cooling was filtered off, washed with MeOH, and recrystallized from ethanol to give the corresponding metal complex.

### 2.3. Nitrogen Gas Adsorption Measurements

The metal complexes were dried at 100 °C for 5 h prior to the measurements. The specific surface areas of the metal complexes were evaluated from the N_2_ adsorption isotherms at 77 K using the Brunauer-Emmett-Teller (BET) method. The pore volumes and sizes were determined using the Barrett-Joyner-Halenda (BJH) method.

### 2.4. CO_2_ Storage Measurements

The CO_2_ uptake of the synthesized metal complexes was measured using an H-sorb 2600 high-pressure volumetric adsorption analyzer (Beijing, China). A sample of the appropriate metal complex (1 g) was degassed in a vacuum oven for 5 h at 70 °C to remove the moisture trapped within the pores. The CO_2_ uptake experiment was carried out several times to optimize the pressure. The CO_2_ adsorption results were reproducible from the experiments that have been carried out at the same conditions.

## 3. Results and Discussion

### 3.1. Synthesis of Metal Complexes

**Figure 1:**
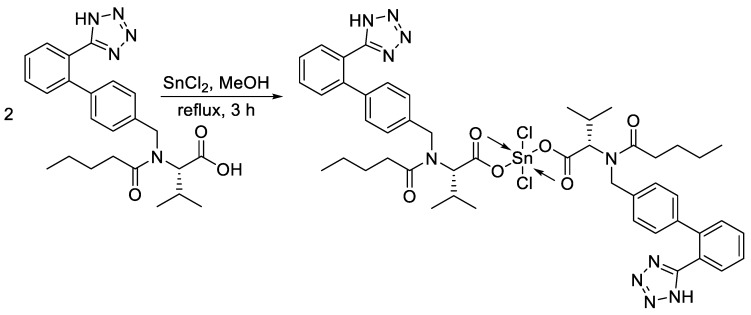
Synthesis of Sn (IV) complex.

**Figure 2:**
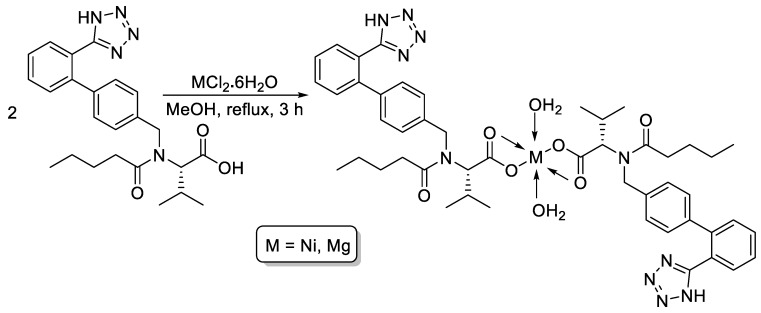
Synthesis of Ni (II) and Mg (II) complexes.

The reaction of valsartan (two mole equivalents) and the appropriate metal chloride (SnCl_2_, NiCl_2_·6H_2_O, or MgCl_2_·6H_2_O) in boiling methanol afforded the corresponding valsartan metal complex (Figure 1 and Figure 2) in excellent yield. The purity of the synthesized complexes was determined by the elemental analyses of carbon, hydrogen, and nitrogen. In addition, the metal content was measured using a flame atomic absorption spectrophotometer. The color, melting point (M.P.), yields, and elemental analyses of the synthesized metal complexes are shown in Table 1.

The elemental compositions of the metal complexes were analyzed using energy dispersive X-ray (EDX), which confirmed the types of elements that each complex contained. The EDX graphs of metal complexes are shown in Figures S1–S3. The synthesized metal complexes were characterized by FT-IR spectra. Table 2 presents the main functional groups (C=O and M–O) within the complexes [39]. The absorption bands for the asymmetric carbonyl groups of the carboxylic moieties within the metal complexes shifted to longer wavenumbers (1732–1735 cm^−1^), compared to that obtained for valsartan (1670 cm^−1^) [40]. Similarly, the symmetric absorption bands for the C=O bonds of the carboxylic groups appeared in the 1470–1474 cm^−1^ region compared to 1442 cm^−1^ for valsartan. The FT-IR spectra of the metal complexes showed new absorption bands that were assigned to *ν*Sn–O (451 cm^−1^), *ν*Ni–O (466 cm^−1^), and *ν*Mg–O (513 cm^−1^) [41,42,43]. The FT-IR spectra of the Ni and Mg complexes showed broad absorption bands at around 3400 cm^−1^ due to the OH group of the water molecules associated with the metal [44]. The difference between the asymmetric and symmetric vibration frequencies of the C=O bonds of the carboxyl groups was in the range of 258–265 cm^−1^, which confirmed the bidentate asymmetry of the metal complexes [45]. The FT IR spectra of valsartan and its metal complexes are shown in in Figures S4–S7.

The electronic spectra of the metal complexes showed a strong absorption band in the 263–264 nm region due to the π→π* electronic transition (Table 2). No d–d transition was observed for either the Sn (IV) or the Mg (II) complex, because they are diamagnetic. The geometry of the Sn and Mg complexes could be sp^3^d^2^-octahedral. However, the electronic spectra of the Ni (II) complex showed an absorption band at 458 nm (21,834 cm^−1^), which is attributed to the d–d transition of the type 3A_2_g (F)→3T_1_g (P) [46]. The UV spectra of valsartan and its metal complexes are shown in Figures S8–S11. The magnetic moment of the Ni complex was approximately 2.5 B.M, which indicates a high-spin sp^3^d^2^-octahedral complex [47]. The molar conductivity measurements confirmed that the synthesized metal complexes were nonelectrolytes. Such results confirm that no ions were liberated in solution, which indicates that anions were present inside the coordination sphere, leading to low molar conductivity (2.2–2.6 µS/cm) [48].

The structures of the synthesized metal complexes were also confirmed from the ^1^H-NMR spectral data (Table 3). The ^1^H-NMR spectra of the complexes showed the absence of a carboxylate proton, which appeared as an exchangeable singlet at 12.63 ppm in the spectrum of valsartan. The deportation of the carboxylic group clearly indicates the formation of O–M bonds. The NH and CH_2_-N protons appeared as singlet signals in the 6.12–6.01 and 4.63–4.59 ppm regions, respectively. The OH protons of water molecules in both the Ni (II) and Mg (II) complexes were not seen, since they overlapped with protons of water in DMSO-d6 [49]. The spectra showed all other protons in the expected chemical shift regions. The ^1^H NMR spectra of valsartan and its metal complexes are shown in Figures S12–S15.

### 3.2. Surface Morphology of Metal Complexes

**Figure 3:**
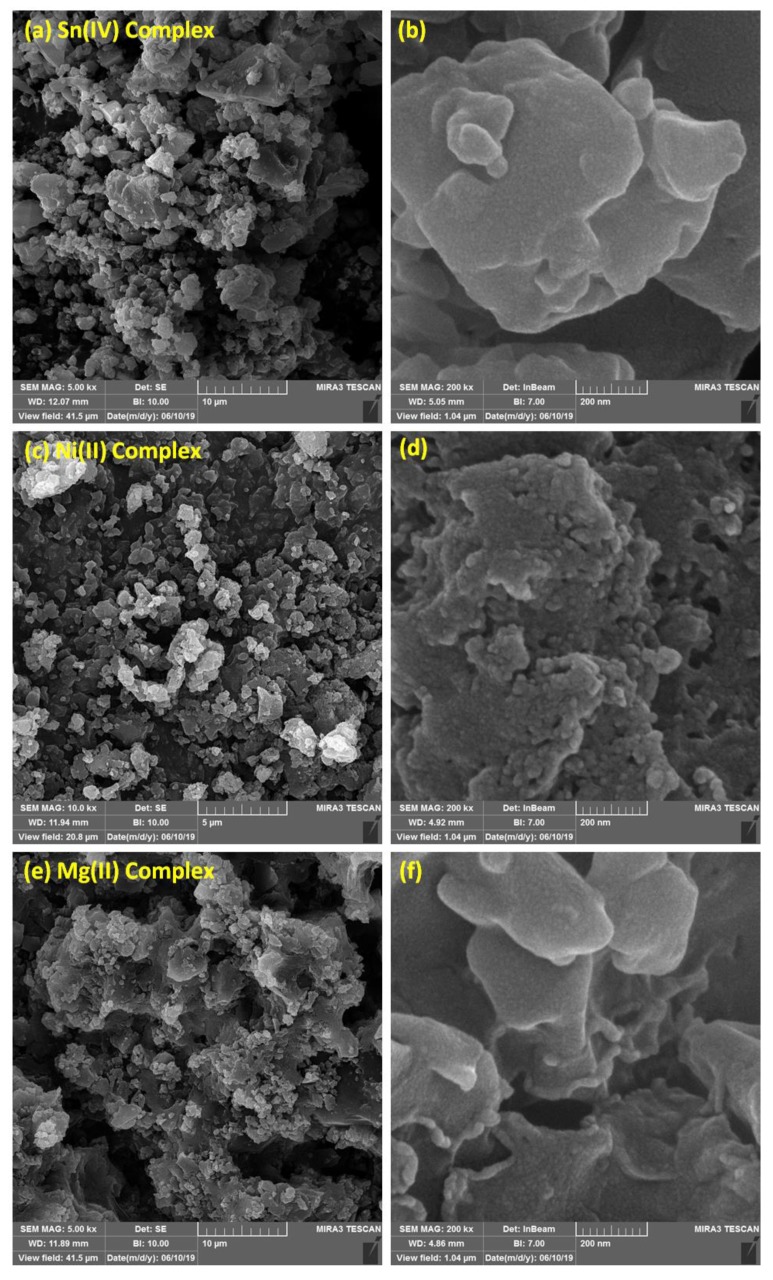
Field emission scanning electron microscopy (FESEM) images of Sn (**a**: 10 μm and **b**: 200 nm), Ni (**c**: 10 μm and **d**: 200 nm), and Mg (**e**: 10 μm and **f**: 200 nm) complexes.

FESEM provides high-resolution, clear, and less-distorted images of the particles of the examined materials. Therefore, the surface morphologies of the synthesized complexes were investigated using FESEM. Figure 3 shows that the surfaces of the metal complexes consisted of very small particles that agglomerated in homogeneous forms at low and high magnification level. The particles had different shapes and diameters, crystals, and some cracks in the cement wall shapes.

### 3.3. CO_2_ Uptake Capacity of Metal Complexes

**Figure 4:**
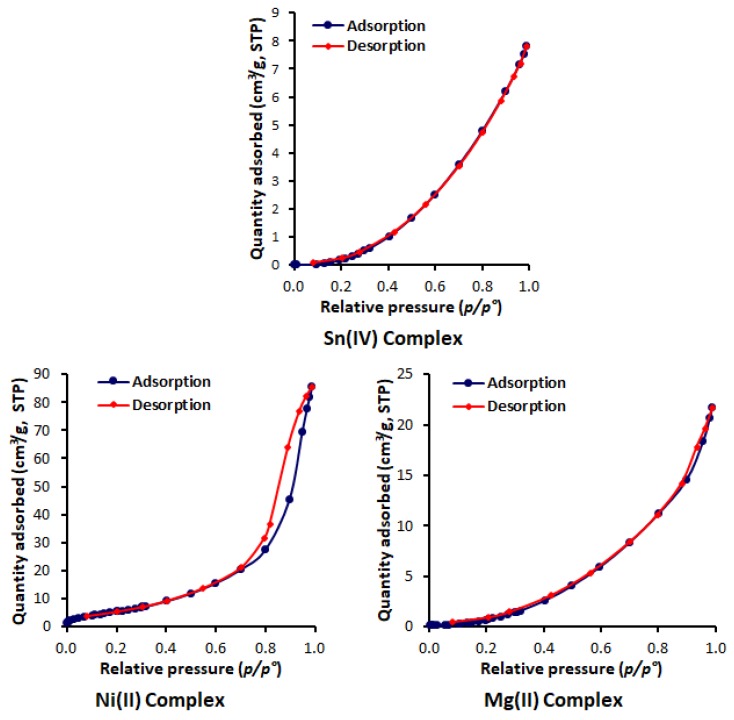
Adsorption-desorption isotherms of N_2_ of metal complexes.

The N_2_ adsorption-desorption isotherms of the synthesized metal complexes are shown in Figure 4. For the Sn and Mg complexes isotherms, the adsorption and desorption branches are completely overlapped, while no overlap was seen for the adsorption and desorption curves related to the Ni complex and no hysteresis loop was generated, as usually happens for mesoporous materials. Based on the IUPAC physisorption isotherm classification, the isotherms of the Sn and Mg complexes are type III, whereas the Ni complex has a type IV isotherm. The sharp bend at the beginning of the curve for the Ni complex is where the completion of monolayer coverage followed by pore condensation is joined by hysteresis due to the increased number of van der Waals forces [50]. For both type III and IV isotherms, no multilayer formation was identified, and both are associated with mesoporous materials. In addition, the adsorbent-adsorbate interactions were relatively weak, and the gas was absorbed on the surface of the complex around the active sites [51,52].

**Figure 5:**
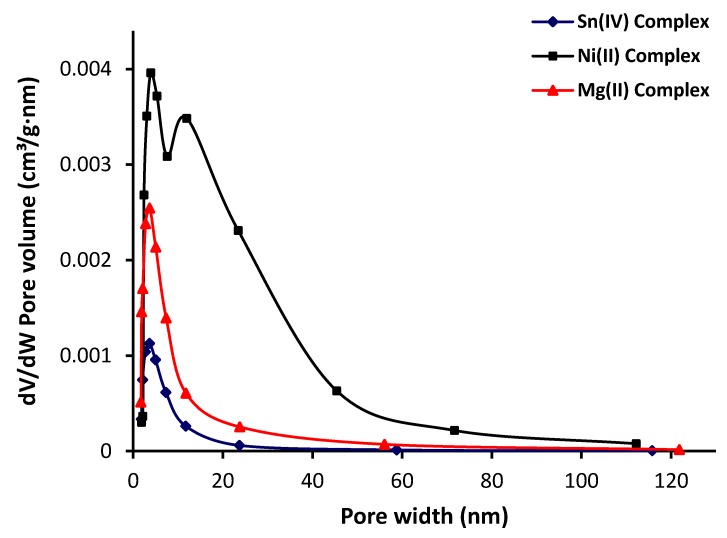
Pore-volume distribution with pore size of metal complexes.

The BET method is the most widely used method to determine the surface area of a porous material in which the physical adsorption of gas on a solid surface is studied. The pore-size distribution and pore volume were calculated from the N_2_ adsorption data using the BJH model [53]. The pore-size distributions of the complexes were obtained by plotting the pore volume (*dV*/*dW*, where *V* and *W* are the volume and diameter of the pores, respectively) versus the pore-size diameter [54]. Figure 5 shows that the pores had diameters between 2 and 120 nm, and that the pore size decreased within increasing pore volume. The pore volume of the Ni (II) complex was higher than those obtained for both the Sn (IV) and Mg (II) complexes. Table 4 shows the surface area (S_BET_), calculated using the BET method; total pore volume, which was evaluated from the single-point adsorption at a relative pressure of 0.95; and the BJH adsorption average pore size. The synthesized metal complexes had small surface areas (15.96–22.75 m^2^/g) and small pores volumes and diameters. The Ni (II) complex had the highest surface area, pore volume, and diameter compared to the other two complexes.

**Figure 6:**
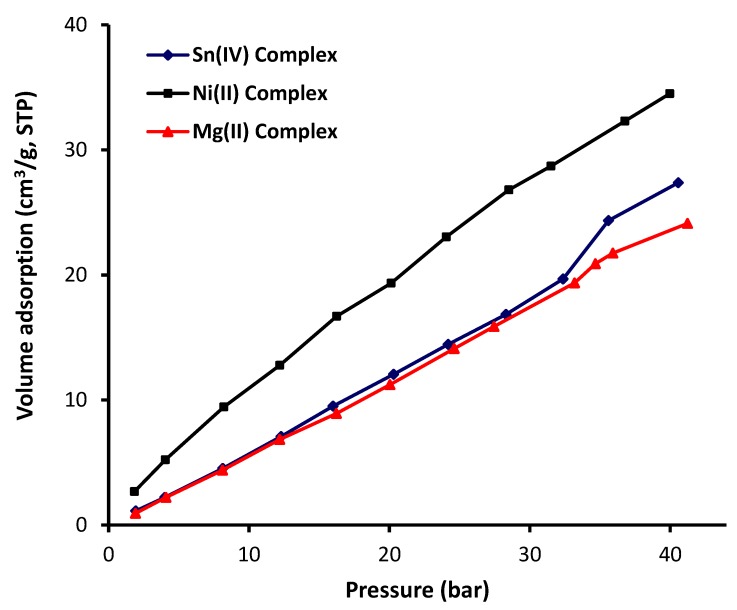
Metal complexes adsorption isotherms of CO_2_.

The CO_2_ adsorption isotherms of the metal complexes measured at 323 K and up to 4 MPa (40 bar) are shown in Figure 6. The metal complexes adsorption isotherms represented in Figure 6 clearly showed that the interaction between the metal complexes and CO_2_ was not favorable. Additionally, the adsorption volume of CO_2_ increased sharply as the pressure reached to 4 MPa. The Ni complex, which had the highest surface area, was the most effective complex toward CO_2_ adsorption (34.51 cm^2^/g) compared to both the Sn (27.37 cm^2^/g) and the Mg (24.11 cm^2^/g) complexes. The behavior of both the Sn and Mg complexes was almost similar until the pressure reached 3.3 MPa (33 bar). However, the performance of the Sn complex was slightly better than that of the Mg complex at a higher pressure. For example, the adsorption of volume at 3.5 and 4 MPa was 24.3 and 27.4 cm^3^/g for the Sn complex and 21.7 and 24.14 cm^3^/g for the Mg complex, respectively. The variation between the Sn and Mg complexes in terms of CO_2_ uptake at a pressures greater than 3.3 MPa (33 bar) depends on the strength of the interaction between the gas molecules and the adsorbent material, but the difference was limited. Table 5 shows the CO_2_ uptake capacity of metal complexes recorded at 323 K and 4 MPa (40 bar).

The CO_2_ uptake capacities of the synthesized metal complexes were comparable to those of polyphosphates containing 1,4-diaminobenzene and telmisartan tin complexes and lower than those obtained in the presence of melamine Schiff bases and polyphosphates containing benzidine as adsorbents (Table 6). However, nanocarbons such as polyacrylonitrile and resorcinol-formaldehyde resin in the presence of a base (potassium hydroxide or potassium carbonate) provided remarkable CO_2_ uptakes of 4.95 and 2.74 mmol/g, respectively, at 25 °C and 100 KPa (1 bar) [23,55]. In the current study, the high pressure used limited the application of the synthesized materials as carbon dioxide storage media. Nevertheless, the materials had some good properties to be tested as additives to inhibit photodegradation of poly (vinyl chloride) upon irradiation with ultraviolet light for long duration.

Heteroatoms (oxygen and nitrogen) and the amide linkage (–CONH–) in valsartan have a high polarity and a nucleophilic nature that would enhance the interaction between CO_2_ and the adsorbent [56,57]. In addition, the metal acts as a Lewis acid that would promote the electrostatic interaction with CO_2_ [58,59]. The van der Waals weak bonds are common in materials that have small pore sizes [60]. The Ni complex is highly acidic, with a high surface area and large pore diameter, compared to the other two metal complexes [61]. Nevertheless, the physisorption phenomena are generally limited due to the metal complexes textural properties.

## 4. Conclusions

Three valsartan complexes were synthesized in high yields using a simple procedure, and their structures were established using various spectroscopic and analytical techniques. The surface morphologies of the metal complexes were inspected using FESEM, which showed different particle shapes and sizes. The surface areas of the metal complexes were relatively small, but that of the Ni complex was the highest. Similar observations were made with pore volumes and diameters. The Ni complex showed the highest affinity toward carbon dioxide capture compared to the Sn and Mg complexes, possibly due to its relatively high surface area compared to those of the other complexes. Additionally, Sn metal is a stronger Lewis acid than the other two metals, which would promote the electrostatic interaction with carbon dioxide. The efficiency of the synthesized metal complexes as carbon capture media is comparable with those reported for some polyphosphates and telmisartan tin complexes, but lower than those reported for melamine Schiff bases and highly aromatic polyphosphates [35,36,37,38].

## Figures and Tables

**Table 1:** Physical properties and elemental analysis of metal complexes.

Complex	Color	M.P. (°C)	Yield (%)	Calculated (Found; %)			
				C	H	N	M
Sn (IV)	off white	147–149	87	54.48 (54.46)	5.39 (5.33)	13.24 (13.23)	11.22 (11.21)
Ni (II)	blue	331–332	90	59.82 (59.82)	6.31 (6.28)	14.55 (14.53)	6.12 (6.09)
Mg (II)	white	157–160	92	62.05 (62.03)	6.52 (6.51)	15.12 (15.07)	2.63 (2.62)

**Table 2:** Fourier transform infrared (FT-IR) spectral data, electronic transition, and conductivity of metal complexes.

Complex	Wavenumber (cm^−1^)				λ (nm)	Transition	Conductivity (µS/cm)
	C=O Sym	C=O Asym	∆v	M–O			
Sn (IV)	1470	1735	265	451	264	π→π*	2.4
Ni (II)	1470	1735	265	466	263, 458	π→π*, ^3^A_2g_ (F)→^3^T_1g_ (P)	2.2
Mg (II)	1474	1732	258	513	263	π→π*	2.6

**Table 3:** Proton nuclear magnetic resonance (^1^H-NMR) spectral data for metal complexes.

Complex	^1^H-NMR (400 MHz: DMSO-d6, δ, ppm, *J* in Hz)
Sn (IV)	7.69 (d, *J* = 8.1 Hz, 4H, Ar), 7.59 (d, *J* = 8.1 Hz, 4H, Ar), 7.09–6.97 (m, 8H, Ar), 6.01 (s, exch., 2H, 2 NH), 4.63 (s, 4H, 2 CH_2_), 4.50 (d, *J* = 7.3 Hz, 2H, 2 CH), 2.28 (m, 2H, 2 CH), 2.09 (t, *J* = 7.5 Hz, 4H, 2 CH_2_), 1.46–1.34 (m, 8H, 2 CH_2_CH_2_), 0.93 (d, *J* = 7.3 Hz, 12H, 4 Me), 0.77 (t, *J* = 7.5 Hz, 6H, 2 Me)
Ni (II)	7.61 (d, *J* = 8.0 Hz, 4H, Ar), 7.53 (d, *J* = 8.0 Hz, 4H, Ar), 7.17–7.95 (m, 8H, Ar), 6.12 (s, exch., 2H, 2 NH), 4.59 (s, 4H, 2 CH_2_), 4.45 (d, *J* = 7.4 Hz, 2H, 2 CH), 2.27 (m, 2H, 2 CH), 2.05 (t, *J* = 7.6 Hz, 4H, 2 CH_2_), 1.49–1.27 (m, 8H, 2 CH_2_CH_2_), 0.90 (d, *J* = 7.4 Hz, 12H, 4 Me), 0.75 (t, *J* = 7.6 Hz, 6H, 2 Me)
Mg (II)	7.68 (d, *J* = 7.9 Hz, 4H, Ar), 7.51 (d, *J* = 7.9 Hz, 4H, Ar), 7.19–7.98 (m, 8H, Ar), 6.03 (s, exch., 2H, 2 NH), 4.61 (s, 4H, 2 CH_2_), 4.41 (d, *J* = 7.5 Hz, 2H, 2 CH), 2.24 (m, 2H, 2 CH), 2.19 (t, *J* = 7.5 Hz, 4H, 2 CH_2_), 1.53–1.37 (m, 8H, 2 CH_2_CH_2_), 0.92 (d, *J* = 7.5 Hz, 12H, 4 Me), 0.75 (t, *J* = 7.5 Hz, 6H, 2 Me)

**Table 4:** Specific surface area and porosity properties of metal complexes.

Complex	S_BET_ (m^2^/g)	Pore Volume (cm^3^/g)	Pore Diameter (nm)
Sn (IV) Complex	16.63	0.011	6.50
Ni (II) Complex	22.75	0.108	12.47
Mg (II) Complex	15.96	0.027	7.635

**Table 5:** Carbon dioxide (CO_2_) uptake capacity at 323 K and 4 MPa (40 bar) of metal complexes.

Complex	CO_2_ Uptake Capacity		
	cm^3^/g	mmol/g	wt%
Sn (IV) Complex	27.37	1.22	5.4
Ni (II) Complex	34.51	1.53	6.8
Mg (II) Complex	24.11	1.07	4.8

**Table 6:** CO_2_ uptake capacity using various adsorbents.

Adsorbent	CO_2_ Uptake		Condition	Reference
	mmol/g	wt%		
Valsartan metal complexes	1.53	6.8	50 °C, 4 MPa	Current work
Melamine Schiff bases	2.33	10	50 °C, 4 MPa	[35]
Polyphosphates containing 1,4-diaminobenzene	1.42	6.0	50 °C, 4 MPa	[36]
Polyphosphates containing benzidine	3.66	14	50 °C, 5 MPa	[37]
Telmisartan tin complexes	1.54	7.1	50 °C, 5 MPa	[38]

## Supplementary Materials

The following are available online at https://www.mdpi.com/1996-1944/13/5/1183/s1, Figure S1: EDX graphs of Sn (IV) complex, Figure S2: EDX graphs of Ni (II) complex, Figure S3: EDX graphs of Mg (II) complex, Figure S4: UV spectrum of valsartan, Figure S5: UV spectrum of Sn (IV) complex, Figure S6: UV spectrum of Ni (II) complex, Figure S7: UV spectrum of Mg (II) complex, Figure S8: FT-IR spectrum of valsartan, Figure S9: FT-IR spectrum of Sn (IV) complex, Figure S10: FT-IR spectrum of Ni (II) complex, Figure S11: FT-IR spectrum of Mg (II) complex, Figure S12: ^1^H-NMR spectrum of valsartan, Figure S13: ^1^HVNMR spectrum of Sn (IV) complex, Figure S14: ^1^H-NMR spectrum of Ni (II) complex, Figure S15: ^1^HVNMR spectrum of Mg (II) complex.
