# Supplementary material for: Synthesis and Use of Valsartan Metal Complexes as Media for Carbon Dioxide Storage
Source: Materials (Basel). 2020 Mar 6;13(5):1183. doi: 10.3390/ma13051183 (PMC7085107; doi:10.3390/ma13051183)
Supplement: Supplementary file 1 [file materials-13-01183-s001.pdf]

materials

Supplementary Materials

Synthesis and Use of Valsartan Metal Complexes as Media for Carbon Dioxide Storage

Alaa Mohammed [1], Emad Yousif [1,*] and Gamal A. El-Hiti [2,*]

[1] Department of Chemistry, College of Science, Al-Nahrain University, Baghdad 64021, Iraq; alaaalqaycy7@gmail.com
[2] Cornea Research Chair, Department of Optometry, College of Applied Medical Sciences, King Saud University, P.O. Box 10219, Riyadh 11433, Saudi Arabia
* Correspondence: emad_yousif@hotmail.com (E.Y.); gelhiti@ksu.edu.sa (G.A.E.-H.); Tel.: +966-11469-3778 (G.A.E.-H.); Fax: +966-11469-3536 (G.A.E.-H.)

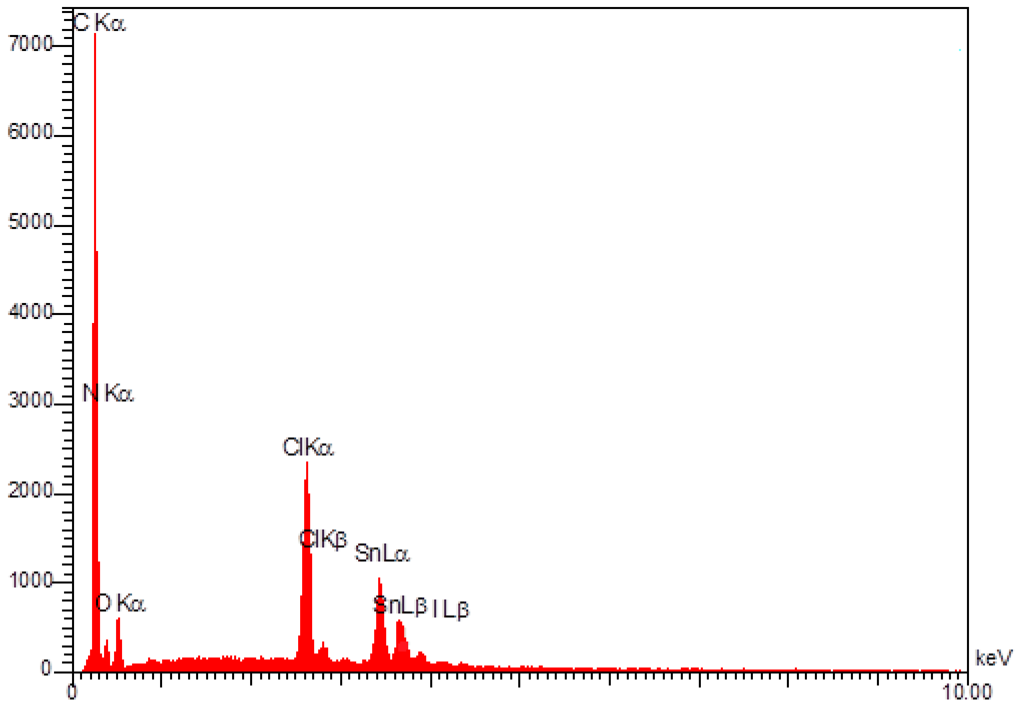

Figure S1. EDX graphs of Sn (IV) complex.

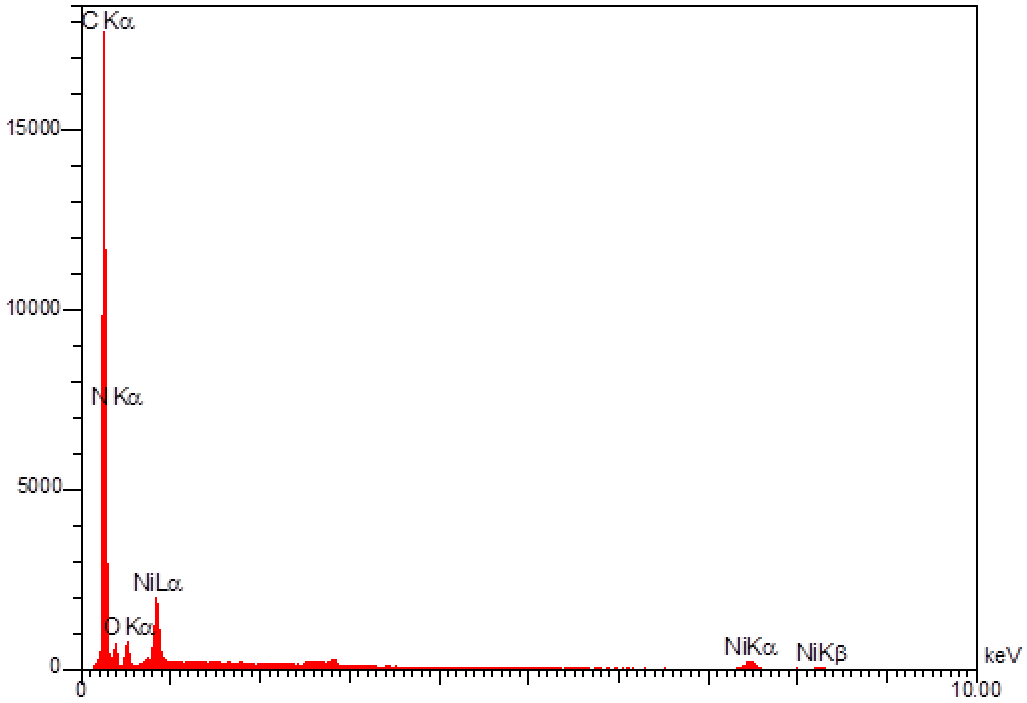

Figure S2. EDX graphs of Ni (II) complex.

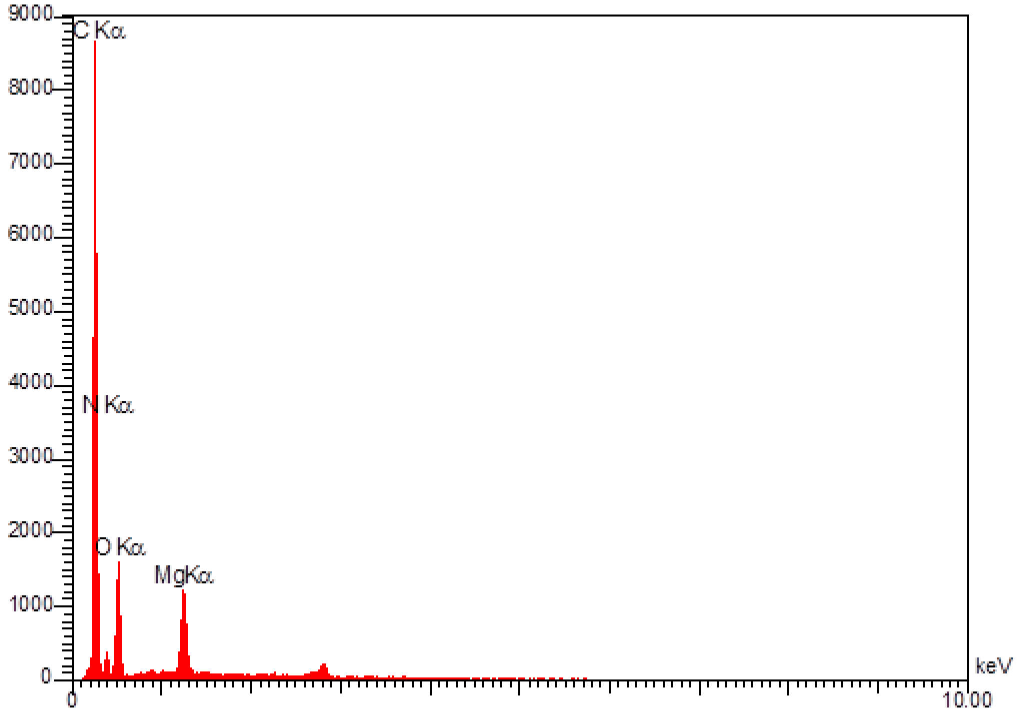

Figure S3. EDX graphs of Mg (II) complex.

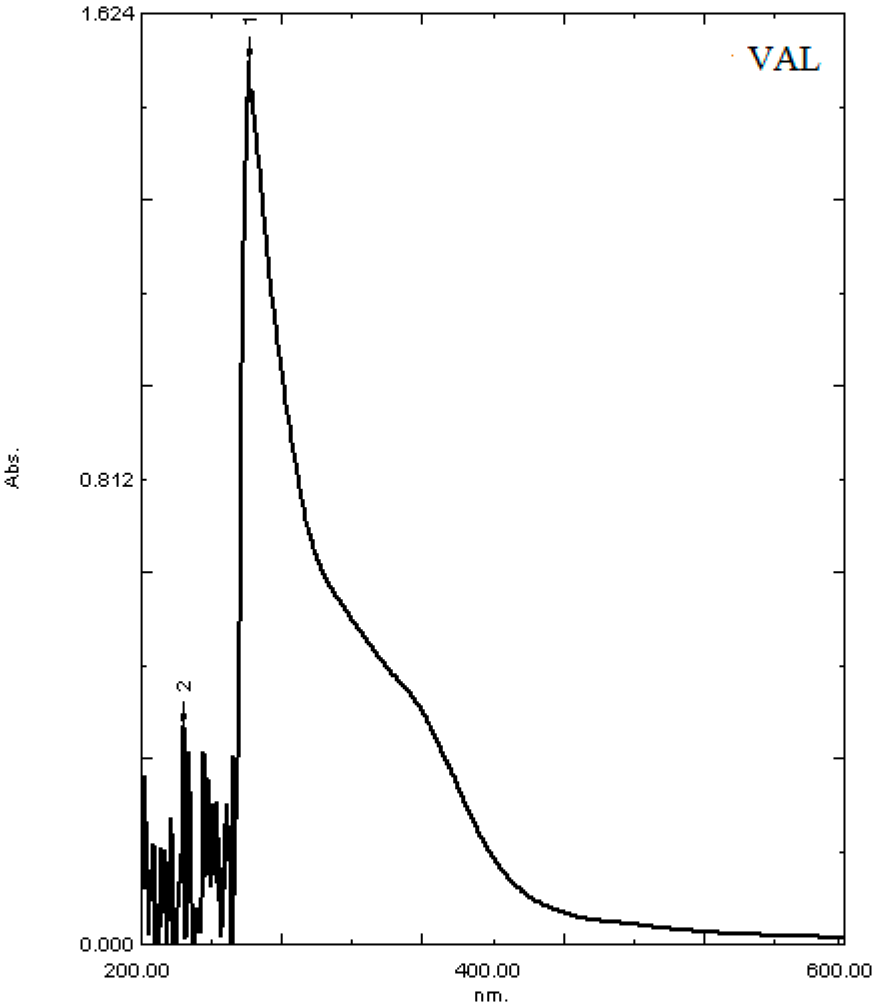

Figure S4. UV spectrum of valsartan.

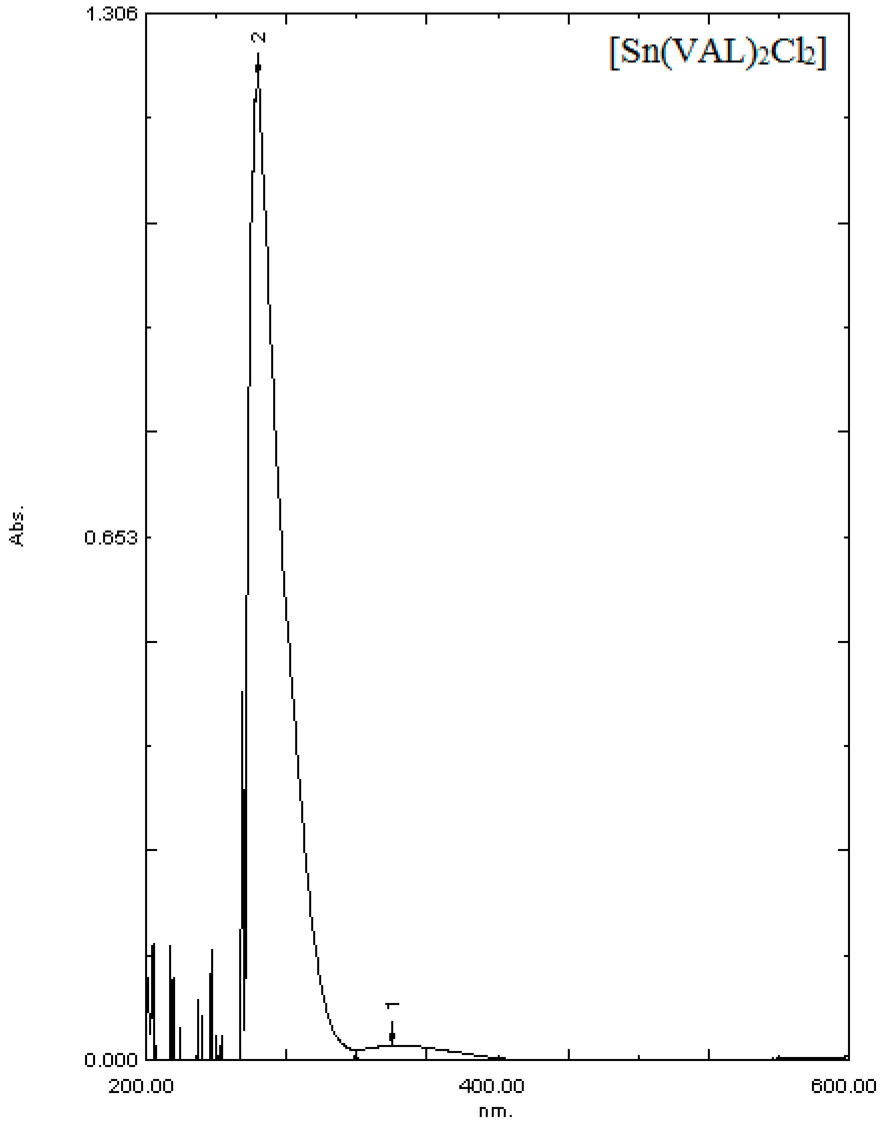

Figure S5. UV spectrum of Sn (IV) complex.

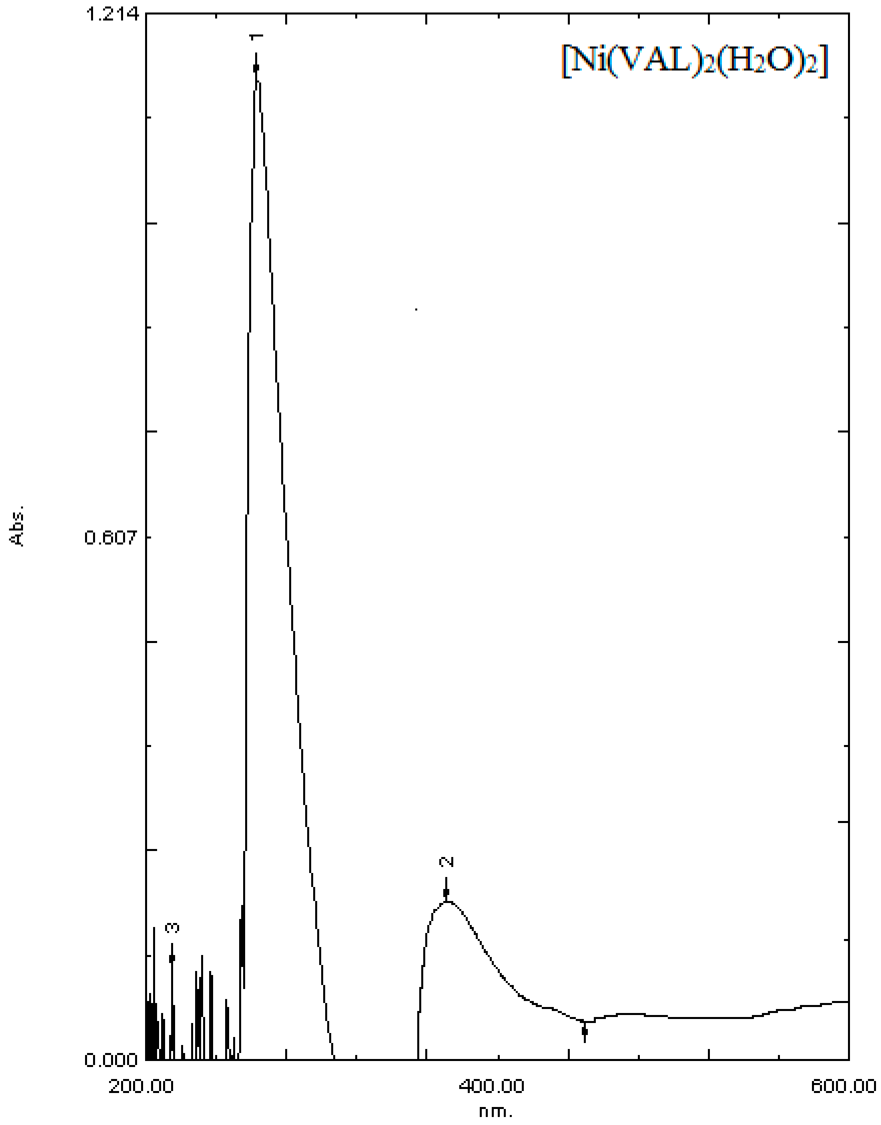

Figure S6. UV spectrum of Ni (II) complex.

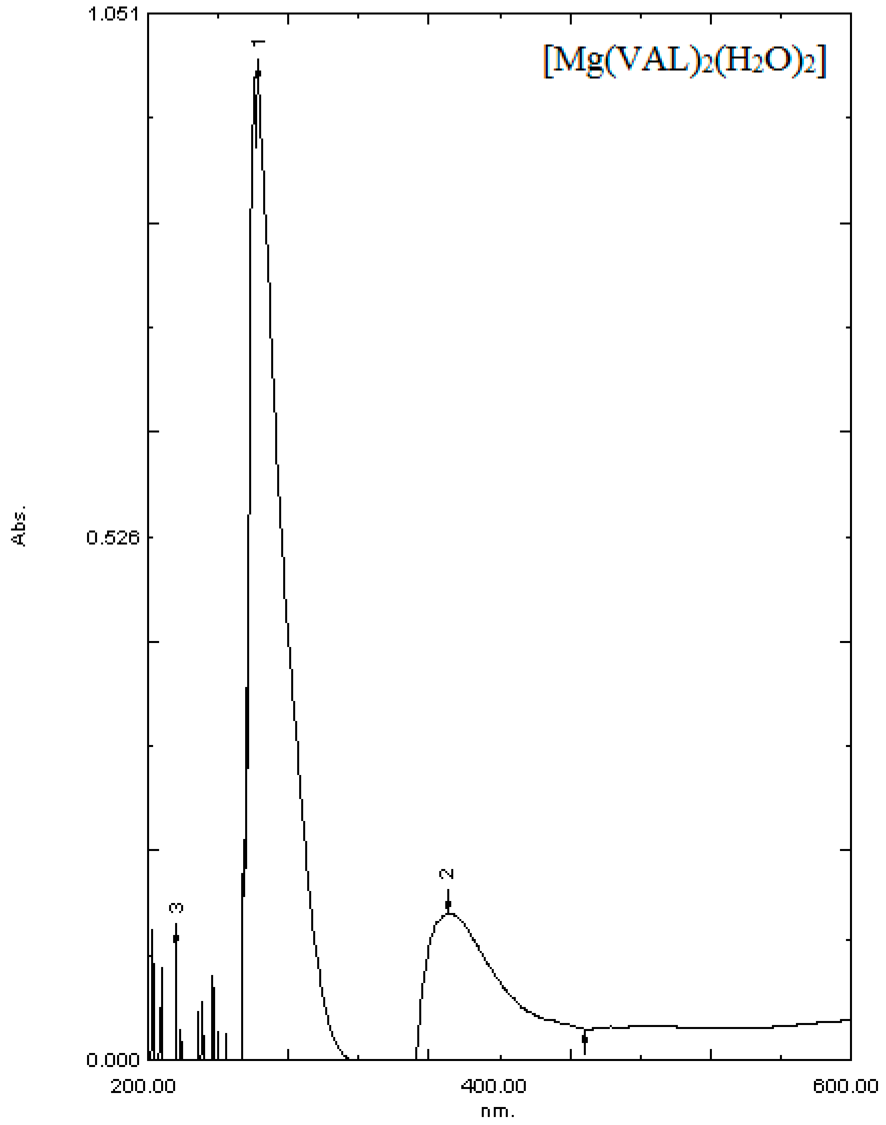

Figure S7. UV spectrum of Mg (II) complex.

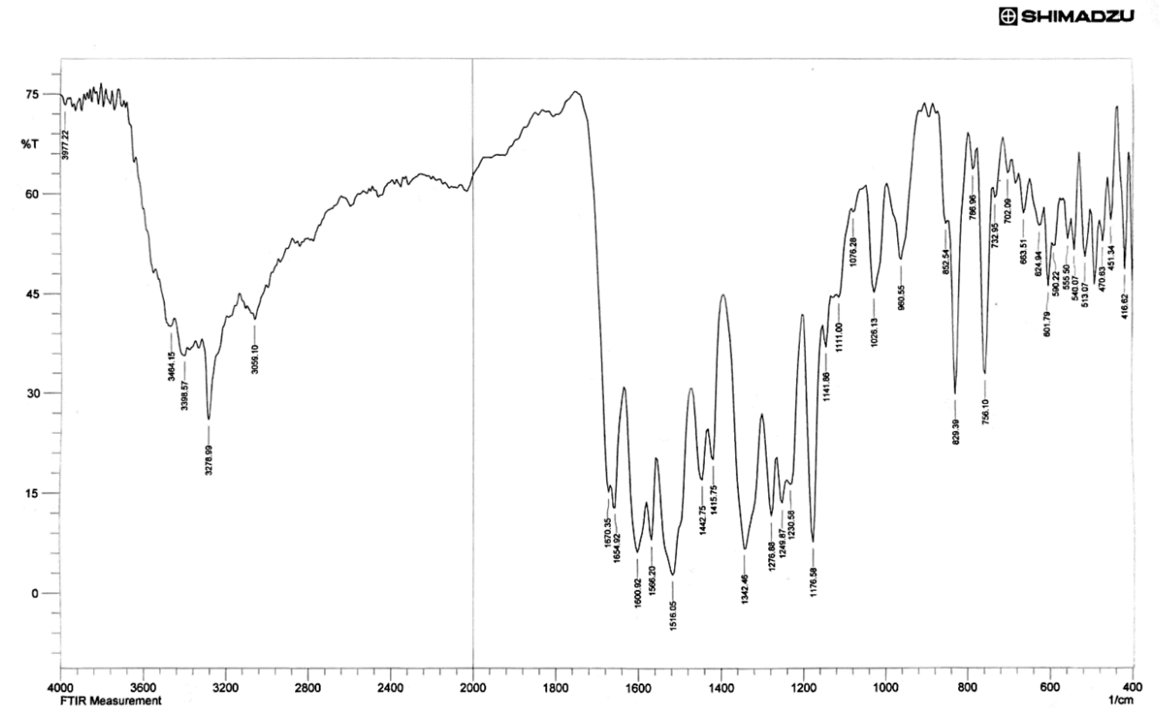

Figure S8. FT-IR spectrum of valsartan.

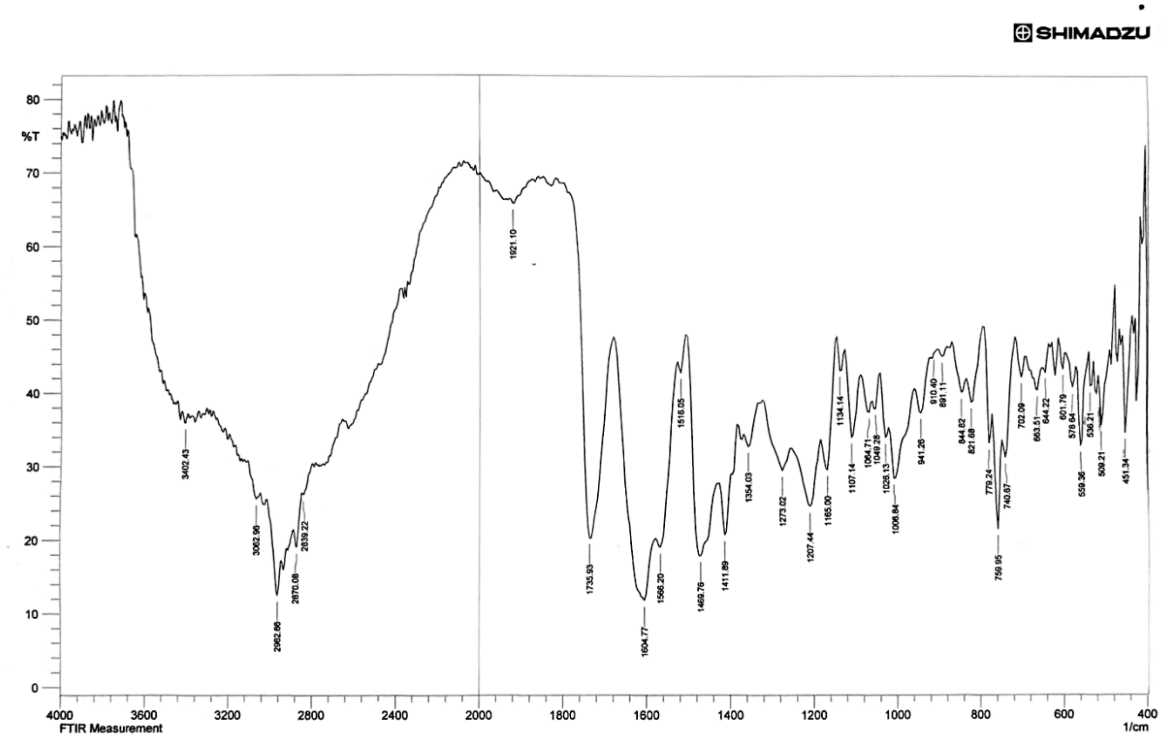

Figure S9. FT-IR spectrum of Sn (IV) complex.

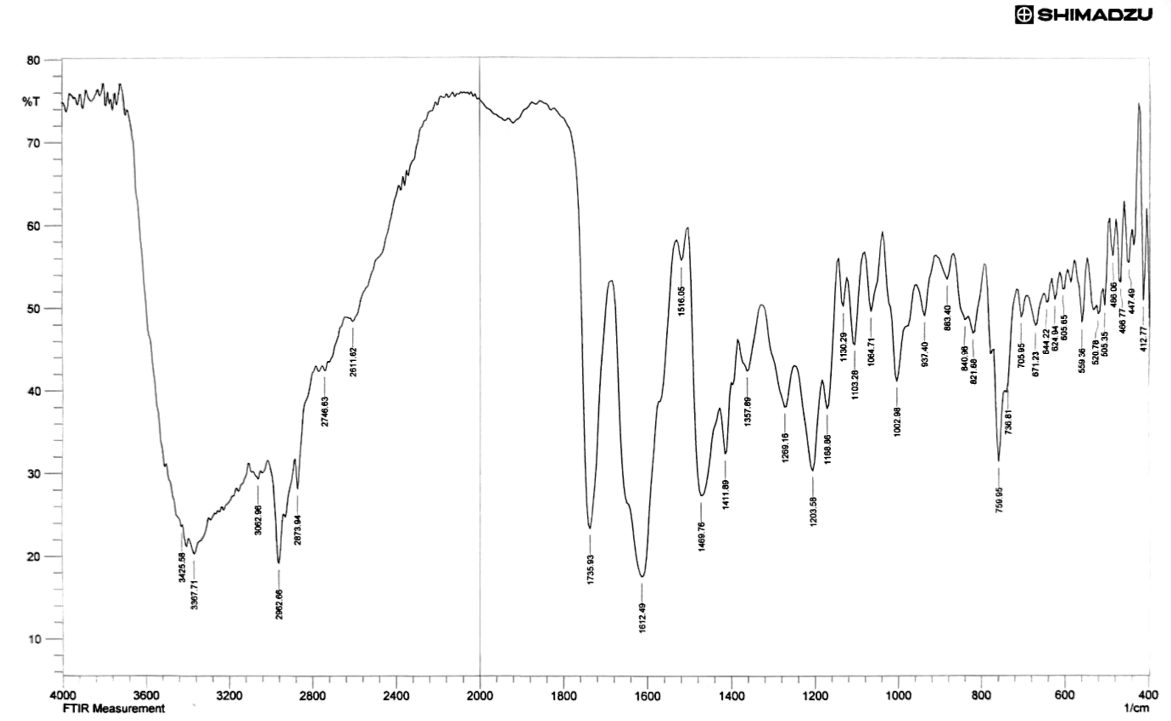

Figure S10. FT-IR spectrum of Ni (II) complex.

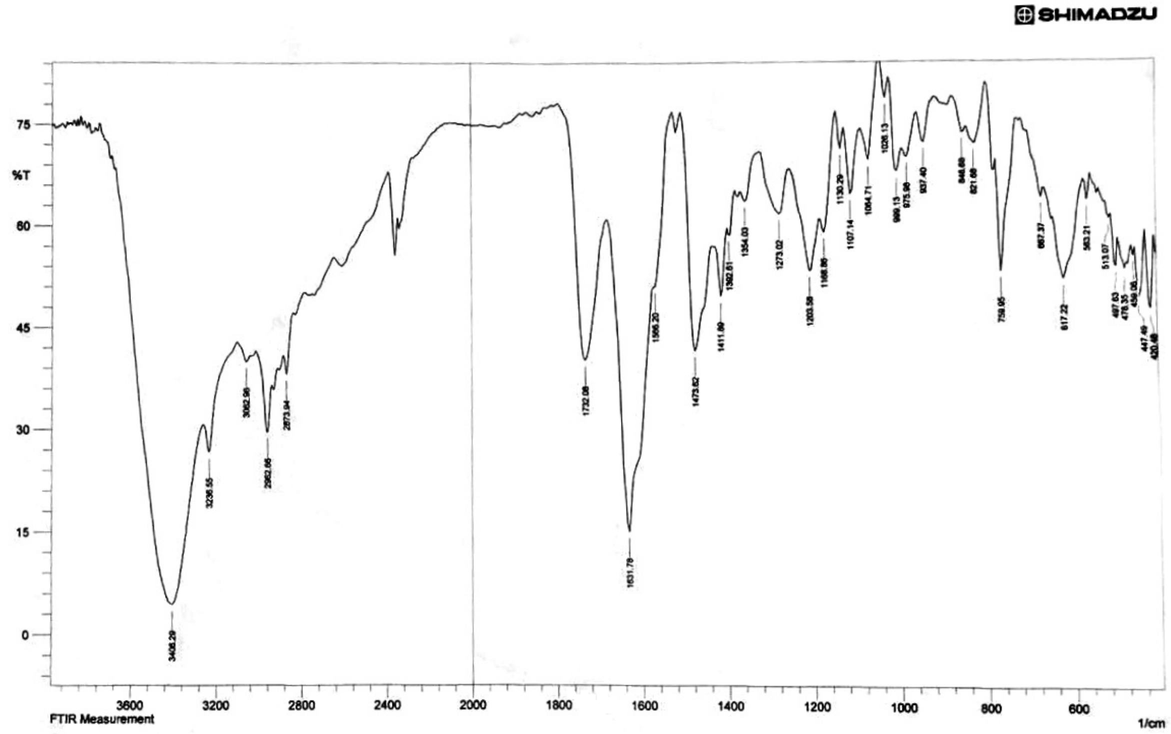

Figure S11. FT-IR spectrum of Mg (II) complex.

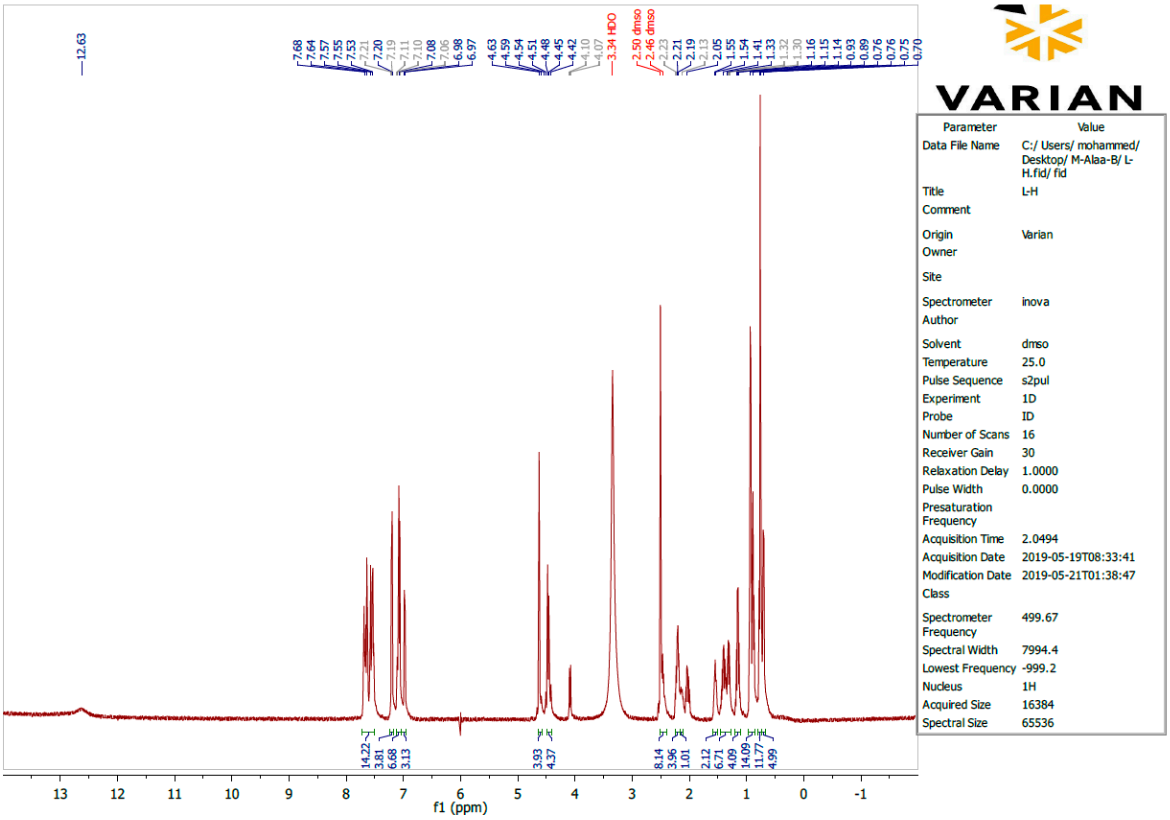

Figure S12. ^{1}H-NMR spectrum of valsartan.

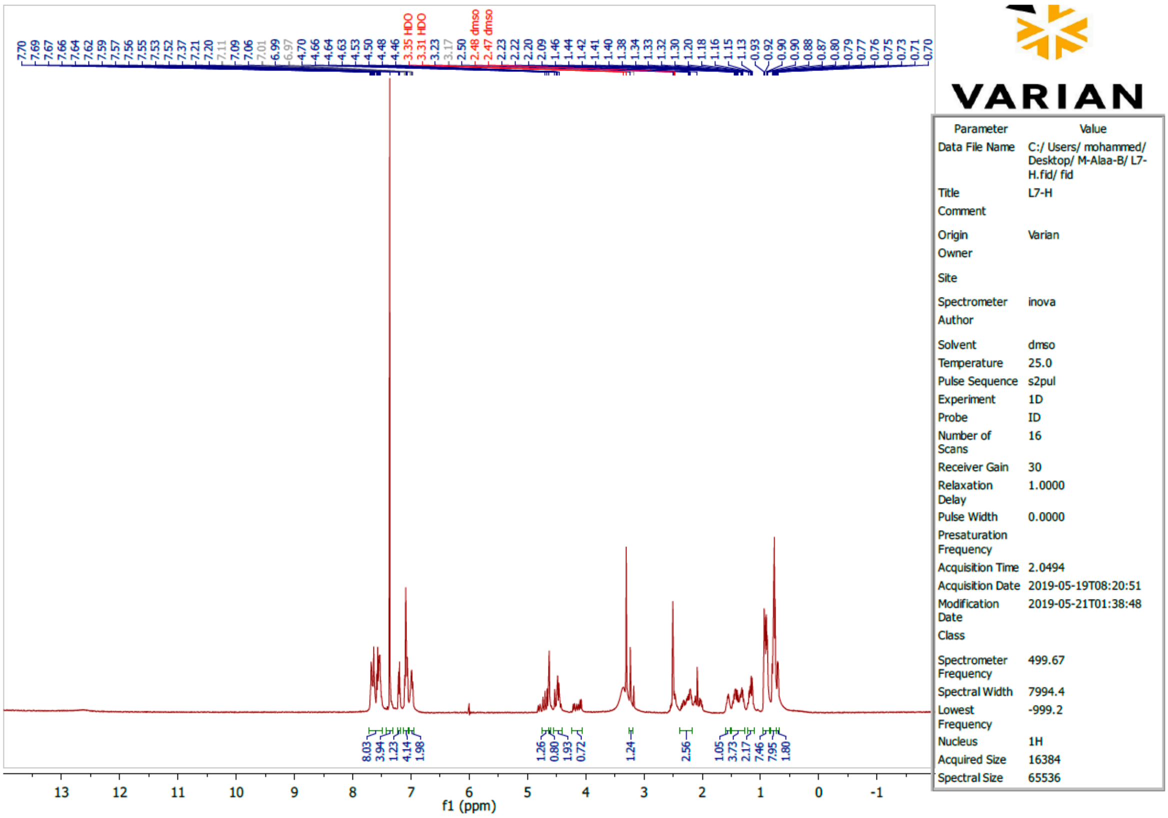

Figure S13. ^{1}HVNMR spectrum of Sn (IV) complex.

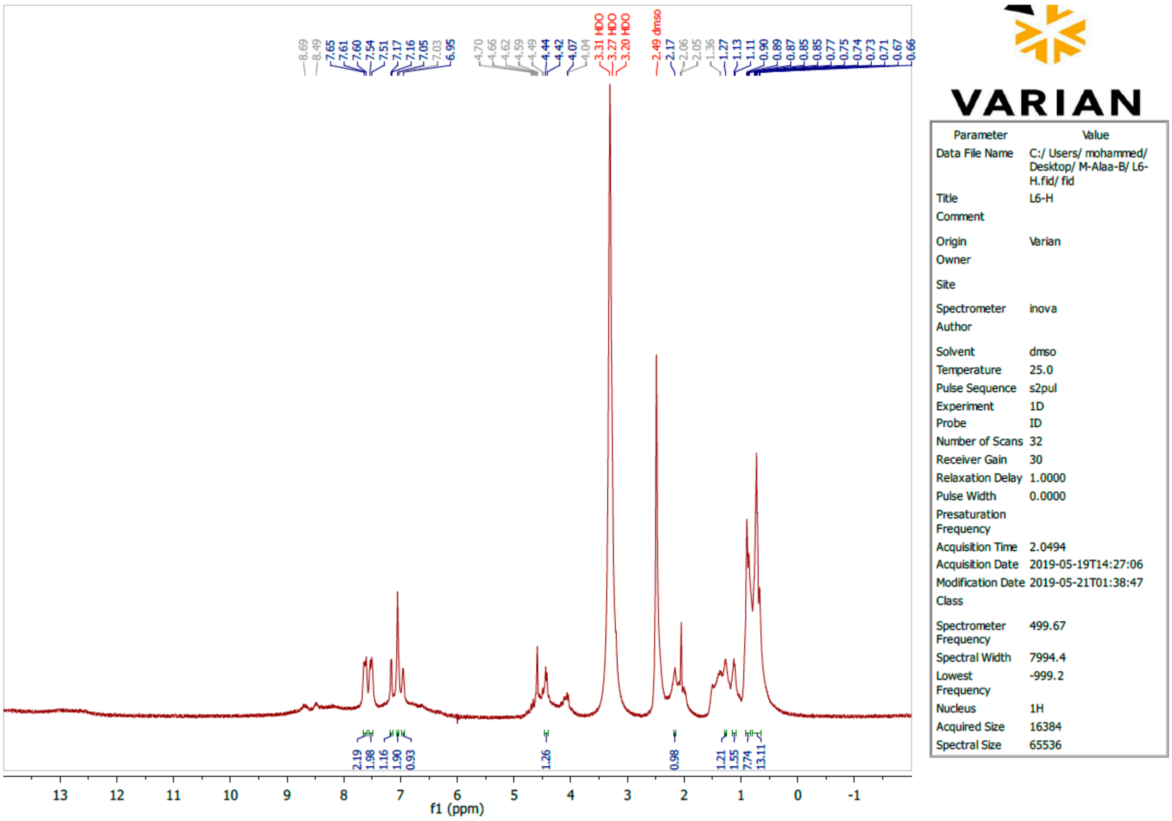

Figure S14. ^{1}H-NMR spectrum of Ni (II) complex.

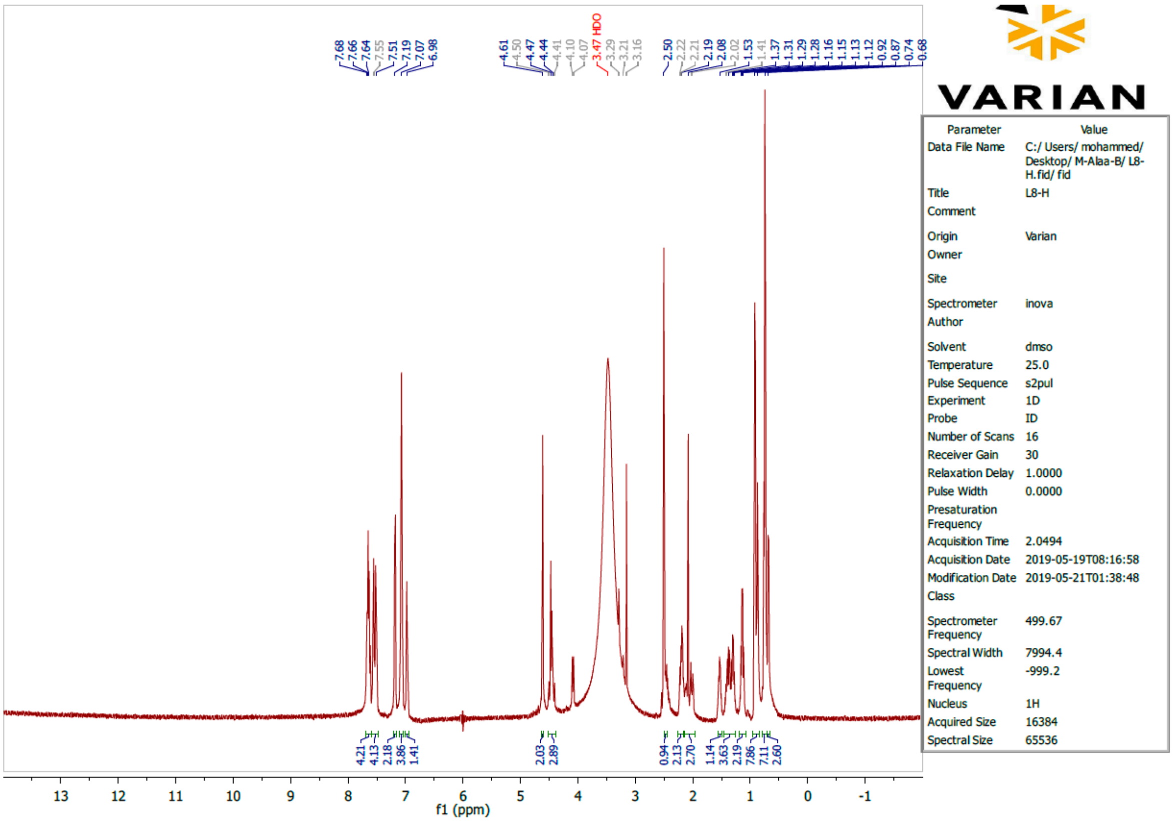

Figure S15. ^{1}HVNMR spectrum of Mg (II) complex.
